# The psychosocial profile of family caregivers of children with chronic diseases: a cross-sectional study

**DOI:** 10.1186/s13030-020-00201-y

**Published:** 2020-10-22

**Authors:** Filiberto Toledano-Toledano, David Luna

**Affiliations:** 1 Evidence-Based Medicine Research Unit, Hospital Infantil de México Federico Gómez, National Institute of Health, Dr. Márquez 162, Doctores, Cuauhtémoc, 06720 México City, Mexico; 2 Comisión Nacional de Arbitraje Médico, Mitla No. 250-8° Piso, esq. Eje 5 Sur (Eugenia). Vertiz Narvarte, 03020, Benito Juárez, Mexico City, Mexico

**Keywords:** Family caregivers, Psychosocial profile, Resilience, Adversity, Psychosocial factors, Cluster analysis, Vulnerability, Well-being, Anxiety, Depression, Parental stress, Caregiver burden

## Abstract

**Background:**

A family caregiver is defined as a person who has a significant emotional bond with the patient; this caregiver is a family member who is a part of the patient’s family life cycle; offers emotional-expressive, instrumental, and tangible support; and provides assistance and comprehensive care during the chronic illness, acute illness, or disability of a child, adult, or elderly person. The objectives of this study were to identify the psychosocial profiles of family caregivers of children with chronic diseases and to establish the relationship between these profiles and sociodemographic variables.

**Methods:**

A cross-sectional study was conducted involving 401 family caregivers of children with chronic diseases at the National Institute of Health in Mexico City. The participants responded to the Sociodemographic Variables Questionnaire (Q-SV) for research on family caregivers of children with chronic disease and a battery of 7 instruments that examined anxiety, caregiver burden, family support, depression, resilience, parental stress, and the World Health Organization Well-Being Index*.*

**Results:**

A hierarchical cluster analysis and its confirmation through a nonhierarchical cluster analysis confirmed two profiles of caregivers of pediatric patients with chronic diseases. Profile 1, called *Vulnerability of family caregivers*, is characterized by high levels of anxiety, depression, parental stress and caregiver burden, accompanied by low levels of family support, resilience, and well-being. Profile 2, called *Adversity of family caregivers*, shows an inverse pattern, with high levels of family support, resilience, and well-being and low levels of anxiety, depression, parental stress and caregiver burden. The sociodemographic characteristics are similar for both profiles, with the exception of the caregiver’s family type. Profile 1 shows more single-parent caregivers, while profile 2 includes more caregivers with a nuclear family. However, the type of family did not reach significance for predicting the caregiver’s profile in a bivariate logistic regression model.

**Conclusions:**

The psychosocial profile of family caregivers of children with chronic diseases can be structured according to their psychosocial characteristics. Although no causal factors were detected that define criteria for belonging to one or another profile, the characteristics identified for each indicate the need for specific and differentiated intervention strategies for families facing adversity, risk and vulnerability during a child’s disease.

## Background

The impact and consequences of care among families of children with chronic diseases are a global public health problem with repercussions for the mental and relational health of the caregivers. Pediatric chronic disease represents a central event that constitutes a major challenge for the family, with physical, psychological, socioeconomic and behavioral effects on patients and their caregivers that translate into vulnerability and decreased quality of life and family functioning [1].

During the course of a child’s chronic disease, family caregivers actively participate in different areas of the child’s care, including assisting with biomedical, physical, rehabilitation, psychological, family, social, and institutional health issues. In addition, caregivers are directly involved in long-term treatments, coordinating health services delivery, and managing the social, financial, and emotional challenges that accompany chronic diseases [2]. Consequently, care tasks may cause a burden that can result in caregiver suffering and loss of health.

Empirical evidence has shown that the lifestyle of family caregivers introduces risks to their physical, mental and social well-being. These risks derive from their daily patterns of time use, which are characterized by a significant burden resulting from childcare that increases as the child grows and from providing full-time parental supervision [3, 4]. Moreover, evidence indicates that women are the main family caregivers and take responsibility for most of the physical tasks related to caring for children’s health [5]. Despite a reported increase in men’s participation in assisting with care in contexts of chronic disease [6], women spend more time than men caring for children [3]. During pediatric chronic illness, the responsibilities of family caregivers include providing physical, psychological, spiritual and emotional support, which can increase their burden [7].

In this context, the family caregiver is defined as a person who has a significant emotional bond with the patient. This can be a family member who is part of the patient’s family life cycle; who offers emotional-expressive, instrumental and tangible support; and who provides assistance and comprehensive care during the chronic illness, acute illness or disability of a child, adult or elderly person [8]. The profile of the caregiver has been defined as a set of demographic, social, cultural and psychological characteristics identified in individuals involved in the long-term care of chronically ill patients [9]. In this regard, the characteristics of family caregivers are studied from two perspectives: sociodemographic and psychosocial [10].

The sociodemographic perspective integrates context variables, demographic characteristics of the family caregiver and patient medical data [10]. The typical sociodemographic profile of the family caregiver is an adult female figure who is married and a homemaker, has basic or low education, belongs to a low socioeconomic stratum, has been the only caregiver of the patient since the onset of the disease, has been taking care of the sick child for more than a year and spends more than 6 h a day doing so [4–6, 11–13] while bearing the financial burden and having unmet medical needs herself [14].

The psychosocial perspective on the family caregiver incorporates personal, family and sociocultural factors associated with the caregiver’s adjustment and adaptation to situations that involve risk, adversity and vulnerability during the course of the chronic disease. Thus, the psychosocial profile of caregivers is characterized by high levels of burden and burnout [7, 15], parental stress and positive adaptation processes despite the loss of health [16], deterioration in family functioning [1], symptoms of depression [17], symptoms of anxiety [18], negative coping styles [19], low levels of resilience [20], little social support [21], optimism [22], and effects on quality of life [1].

The sociodemographic characteristics of caregivers make them an at-risk population characterized by the situations of adversity and vulnerability that affect families of children with chronic diseases. In the current research literature, there are no mechanisms for systematic measurement and evaluation to identify the family caregiver’s state of health of and to perform relevant actions [22, 23]. That is, the physical illness of the patient has consequences in terms of an increase in new cases related to public mental health that require medical attention, and the quality of the attention given to the patient who requires care is reduced. In addition, most researchers have focused on studying family caregivers of adult patients, neglecting the psychosocial profile of those who care for pediatric patients. Therefore, the objectives of this study were to identify the psychosocial profiles of family caregivers of children with chronic diseases and to describe the relationship between these profiles and sociodemographic variables.

## Methods

### Participants

In this cross-sectional, descriptive, and ex post facto study, a nonprobabilistic convenience sampling technique was used at the Hospital Infantil de México Federico Gómez National Institute of Health. The inclusion criteria were that the caregiver was in a parenting role, had a child with a chronic disease that required highly specialized hospital treatment, and had provided informed consent. A total of 466 caregivers were invited to participate, of whom 50 (10.72%) did not wish to participate. The elimination criteria were requesting the withdrawal of their study data or partially completing the instruments. Based on these criteria, 416 voluntary participants were recruited.

### Instruments

*Sociodemographic variables questionnaire (Q-SV) for research on family caregivers of children with chronic diseases* [10]. The questionnaire contains 20 questions that evaluate social, family, and clinical variables: the age and gender of the patient and caregiver, diagnosis, time hospitalized and time since diagnosis, the caregiver’s family relationship with the patient (mother, father, other family member), education level (no schooling, primary, secondary, preparatory, bachelor’s degree, postgraduate), occupation (homemaker, manual laborer, merchant, employee, student, pensioner, unemployed), marital status (married, living with significant other, separated, divorced, single mother or father, widowed), number of years with partner, number of children, type of family (nuclear, semiextended, extended, single parent, living with another family), family life stage (with small children, with school-age children, with adult children), social support networks (family, friends, church, institutions, government), religion (Catholic, Christian, none), and monthly income.

*The Beck Depression Inventory BDI-II* Second Edition (BDI-II [24];), validated in family caregivers of children with chronic diseases by Toledano-Toledano and Contreras-Valdez [8]. This self-report instrument consists of 21 items that measure symptoms of depression. Participants respond using a four-point scale (0 to 3), with higher scores indicating more severe depressive symptomology. The alpha coefficient was α = .91.

*The Beck Anxiety Inventory* (BAI [25];), validated in a Mexican population by Robles et al. [26]. The BAI is a multiple choice questionnaire with 21 items. It comprises four factors - subjective anxiety (8 items), neurophysiological anxiety (7 items), autonomic anxiety, and panic (3 items each) - which explain 56% of the variance. It has an internal consistency of α = .93.

*The Mexican Resilience Measurement Scale* (RESI-M [27];), validated in family caregivers of children with chronic diseases by Toledano-Toledano et al. [20]. The RESI-M is a 43-item questionnaire answered using a Likert scale with 4 response options ranging from strongly disagree (1) to totally agree (4). It includes 5 factors - strength and self-confidence (19 items), social competence (8 items), family support (6 items), social support (5 items), and structure (5 items) - which explain 43.60% of the variance. The alpha coefficient was α = .95.

*The Zarit Burden Interview* (ZBI [28];), validated in a Mexican population [29]. This self-report instrument consists of 22 items (α = .90) with five response options (0, *never*, to 4, *always*). Higher scores indicate a greater level of burden. The ZBI is a 22-item questionnaire answered using a Likert format with 5 answer options ranging from never (0) to always (4). It comprises three factors - impact of care (13 items), interpersonal relationship (6 items), and self-efficacy expectations (3 items) - which explain 50.4% of the variance. The alpha coefficient was α = .90.

*Parental Stress Scale* (PSS [30];), validated Spanish version [31]. This instrument includes 12 items answered using a Likert format with five response options ranging from strongly disagree (1) to totally agree (5). It comprises two factors - rewards (5 items, α Cronbach = 0.76) and stressors (7 items, α Cronbach = 0.77) - which explain 33.5% of the variance. The alpha coefficient was α = .90.

*Family Support Questionnaire* (FSQ [32];). The FSQ is a one-dimensional 17-item scale answered using a Likert s with four response options ranging from never (1) to always (4). The alpha coefficient was α = .95.

*The WHO (Ten) Well-Being Index* (WHO-TWBI [33];). This one-dimensional scale comprises 10 items answered using a Likert format with four response options ranging from never (0) to all the time (4). The WHO-TWBI was adapted linguistically for the current study using the translation-retranslation strategy. The scale is based on the instrument by Bech et al. [33], which contains 9 items with four Likert-type response options (0, never, to 3, all the time). A higher score indicates greater psychological well-being. The alpha coefficient was α = .90.

The scores were interpreted according to pre-established criteria for the BAI [25], BDI-II [8], ZBI [29], PSS [31], and FSQ [32] scales. To interpret the remaining instruments (RESI-M, WHO-TWBI), quartiles were calculated, and possession of the attribute was measured as low (≤ quartile 1), medium low (> quartile 1 ≤ median), medium high (> median ≤ quartile 3), and high (> quartile 3).

### Procedure and ethical considerations

The protocol of the present study was approved by the Ethics and Biosafety Committee of the Hospital Infantil de México Federico Gómez National Institute of Health, Research protocol: HIM-2013-019-SSA.1141. This study adhered to the ethical rules and considerations for research with humans currently in force in Mexico [34] as well as to those outlined by the American Psychological Association [35]. Participation in this study was voluntary. Prior to completion, the participants were informed of their rights as outlined in the Helsinki Declaration [36].

All participants were provided with information regarding the study’s objective and their research rights, particularly regarding the fact that there were no consequences if they decided not to participate. Data collection was performed by trained personnel in the Evidence Based Medicine Research Unit at the National Institute of Health under the direction of the first author of this study. The data collection process lasted approximately 5 months in 2018 and took place in the rooms of the hospitalized children and in the waiting rooms of the different medical services of the institution. The researchers met with each family caregiver, provided information about the study, informed participants of their research rights, and gave them the informed consent form. The battery of tests was administered individually.

### Data analysis

#### Cluster analysis

To identify the psychosocial profiles of the caregivers, cluster analysis was used. The cluster variable was the scores on all the original scales of the instruments described above except the sociodemographic data questionnaire, due to the sensitivity of cluster analysis to the presence of outliers [37]. Initially, univariate and multivariate outliers were detected, and the data of those participants whose scores on the instruments were > |3| standard deviations from the mean or whose Mahalanobis distance showed a *p* <  0.001 [38]. To determine the optimal number of clusters, a hierarchical cluster analysis was performed by calculating the Euclidean square distance, and the Ward clustering method was used to detect the hierarchical structure [39]. The dendrogram and agglomeration coefficient were analyzed. Then, a confirmatory cluster analysis was performed [40] using a nonhierarchical procedure, allowing the procedure to randomly select the initial centroids. To evaluate the correspondence between the results of the hierarchical and nonhierarchical methods, the gamma, tau-b, tau-c, and d coefficient of Somer were calculated [41]. Prior to the final confirmation of the clusters, they were analyzed for their conceptual coherence.

#### Validation of the cluster analysis solution

Once the clusters were defined, the statistical significance of the differences between them was analyzed. Two-tailed t-tests were performed for independent groups using Cohen’s d as a measure of effect size, considering a small, medium and large effects at d ≥ .20, .50, and .80, respectively [42].

#### Description of the clusters

The psychosocial characteristics of the clusters were analyzed by interpreting the scores of the instruments in categories and performing χ2 tests of independence. When the results were significant, Pearson’s standardized residuals were calculated as a post hoc test [43], and Cramer’s V was calculated as an index of the strength of the association between variables [44]. The strength of the association between variables was interpreted trivially, with absolute values ​​less than .10 interpreted as nonexistent, .11 to .29 as low, .30 to .49 as medium and ≥ .50 as high [45]. Descriptive statistics and frequency analysis were used to determine sociodemographic characteristics. Tests of independence (*t* test and χ2) were used to detect differences or associations between these characteristics and the clusters.

#### Predictive model of caregivers’ psychosocial profiles

A predictive model was estimated using a binary logistic regression with the forward selection technique based on the Wald statistic. The response variable was membership in the resulting clusters. The predictor variables were chosen among the sociodemographic variables, and the criterion for incorporation into the model was a difference or association with the clusters with a *p* <  0.10. The model was validated by evaluating the null hypothesis, and its goodness of fit was determined using the omnibus and Hosmer-Lemeshow tests. The percentage of correct classification of cases and Nagelkerke’s *R*^2^ coefficient of determination were also calculated.

SPSS v.24, IBM, Inc., Chicago, USA, was used for all analyses, and a result was considered significant with *p* ≤ .05.

## Results

### Cluster analysis

The data of 15 participants were eliminated due to the presentation of univariate or multivariate outliers; therefore, the cluster analysis was conducted with the data of the remaining 401 participants (see Table 1).

**Table 1:** Sociodemographic variables for each cluster

Variable	Cluster 1 (*n* = 201)	Cluster 2 (*n* = 200)	***p***
Caregiver gender			0.71
Female	162 (40.4)	164 (40.9)	
Male	39 (9.7)	36 (9)	
Caregiver age +	31.48 (7.74)	32.10 (8.26)	0.44
Civil status			0.08
Married/free union	153 (38.1)	166 (41.4)	
Not married	48 (12)	34 (8.5)	
Economically remunerated activity			0.12
Yes	62 (15.5)	48 (12)	
No	139 (34.7)	152 (37.8)	
Education level			0.40
No education	6 (1.5)	5 (1.2)	
Basic	133 (33.2)	117 (29.2)	
Middle	47 (11.7)	59 (14.7)	
Higher	15 (3.7)	19 (4.8)	
Religion			0.24
Yes	183 (45.8)	189 (47.2)	
No	18 (4.2)	11 (2.8)	
Parental role			0.87
Mother	166 (41.4)	164 (40.9)	
Father	35 (8.7)	36 (9)	
Type of family			0.03
Single parent	39 (9.8)^a^	22 (5.5)	
Nuclear	92 (22.9)	111 (27.7)	
Semiextended	37 (9.2)	27 (6.7)	
Extended	17 (4.2)	26 (6.5)	
Living with another family	16 (4)	14 (3.5)	
Family life cycle			0.77
With small children	65 (16.2)	62 (15.5)	
With adolescents or older children	136 (33.9)	138 (34.4)	
Monthly income			0.63
< 1 MS	124 (30.9)	117 (29.2)	
= 1 MS	45 (11.2)	44 (11)	
V > 1 MS	32 (8)	39 (9.7)	
Social support networks			0.11
Family	163 (40.6)	177 (44.1)	
Institutions	27 (6.7)	16 (4)	
Others	11 (2.8)	7 (1.8)	
Number of children +	2.25 (1.20)	2.37 (1.17)	0.61
Patient’s gender			0.58
Female	98 (24.5)	92 (22.9)	
Male	103 (25.7)	108 (26.9)	
Patient’s age +	6.04 (4.88)	5.93 (5.23)	0.81
Diagnosis			0.84
Oncological	149 (37.2)	150 (37.4)	
Other	52 (13)	50 (12.5)	
Time since diagnosis			0.30
< 3 months	47 (11.7)	58 (14.5)	
3 to 12 months	89 (22.2)	75 (18.7)	
> 12 months	65 (16.2)	67 (16.7)	
Hospitalization time			0.55
< 1 week	127 (31.7)	132 (32.9)	
> 1 week	74 (18.4)	68 (17)	

The variables indicated with + were analyzed with t tests and are shown as M (SD); the others were analyzed with χ^2^ tests of independence and are shown as N (%). *MS* minimum salary, USD$ 132.76 monthly. ^a^ frequency greater than chance

The analysis of the dendrogram obtained through the hierarchical analysis indicated a two- or three-cluster solution. The agglomeration coefficient based on the grouping stages showed an increase of three to two clusters (C = 28,543.04) and of two to one cluster (C = 86,471.58); therefore, a two-cluster solution was chosen. In the nonhierarchical analysis with two clusters, convergence was reached in the sixth iteration. The coherence between the results of both methods was high, gamma = 0.99, tau-b = 0.79, tau-c = 0.78, d of Somer = 0.79, which indicated the adequacy of the two-cluster solution. The conceptual analysis showed coherence between both clusters.

Given these results, the subsequent analyses were performed using the solution and assignment determined by the hierarchical method.

### Validation of the cluster analysis solution

Significant differences between the clusters were detected for the means of the scores obtained for the evaluated psychosocial variables, which also showed a large effect size (Table 2).

**Table 2:** Differences between clusters in the scores obtained for psychosocial variables

	Cluster 1 (*n* = 201)	Cluster 2 (*n* = 200)			
	M (SD)	M (SD)	T (399)	***p***	|d|
Anxiety	20.55 (12.30)	6.74 (5.41)	14.53	< 0.001	1.45
Caregiver burden	29 (9.44)	15.56 (7.02)	16.15	< 0.001	1.61
Family support	56.15 (9.93)	63.86 (6.15)	−9.32	< 0.001	0.93
Depression	18.56 (8.64)	8.41 (6.74)	13.11	< 0.001	1.31
Resilience	126.13 (12.48)	142.46 (14.24)	−12.20	< 0.001	1.22
Parental stress	22.11 (6.97)	16.68 (5.16)	8.86	< 0.001	0.88
Wellness	15.54 (4.05)	21.26 (4.01)	−14.20	< 0.001	1.42

*M* Mean, *SD* Standard deviation; |d|: absolute value of Cohen’s d

### Description of the clusters

Table 3 shows the interpretation by cluster of the scores obtained for the psychosocial variables evaluated. In all cases, the indicated category showed a frequency of occurrence that was greater than chance. These data indicate that cluster 1 is characterized by the presence of higher levels of anxiety, caregiver overload, depression, and parental stress accompanied by lower levels of family support, resilience, and well-being. The opposite occurs with cluster 2, which presents higher levels of family support, resilience, and well-being and lower levels of anxiety, caregiver overload, depression, and parental stress. These contrasting characteristics allow for the interpretation of a profile of caregivers in a vulnerable situation (cluster 1) and a profile of caregivers with strengths (cluster 2).

**Table 3:** Possession level of the psychosocial variables evaluated by cluster

	Cluster 1 (*n* = 201)	Cluster 2 (*n* = 200)	χ^2^	*p*	V
Anxiety	Moderate to severe	Minimum to slight	142.36	< 0.001	0.59**
Caregiver burden	Light	Absent	9.16	0.01	0.15**
Family support	Null to medium-low	Adequate	85.47	< 0.001	0.46**
Depression	Moderate to severe	Minimal	107.21	< 0.001	0.51**
Resilience	Low	High	115.78	< 0.001	0.53**
Parental stress	Medium-high to high	Low to medium-low	63.70	< 0.001	0.39**
Wellness	Low to medium-low	Medium-high to high	134.83	< 0.001	0.58**

V: Cramer’s V; **: *p* < 0.001

The sociodemographic characteristics of each cluster are shown in Table 3. The analysis showed that in cluster 1, there were a greater number of single-parent families, while in cluster 2, the most common type of family was nuclear (χ2 = 6.57; Cramer’s V = 0.12). However, the strength of the association among variables was low. The other sociodemographic variables did not show differences between clusters or in strength of association. In both clusters, the population without paying work is approximately 70%, which raises doubts about how members of this population obtain resources to subsist. Additional evidence showed that in single-parent families, the caregiver has a job (χ2 = 8.72; Cramer’s V = 0.14), and unemployed caregivers receive family support (χ2 = 20.29; Cramer’s V = 0.22) with a frequency higher than the level of chance. However, again, the strength of association among variables was low.

### Predictive model of the psychosocial profile of caregivers

The response variable was that belonging to cluster 1, identified as the profile of vulnerable caregivers. The predictor variables were the marital status of the caregiver and the type of family. The family type variable was recoded by integrating the “semiextended”, “extended” and “with another family” into the “other” category. This was done because the occurrence of these categories did not exceed the frequency expected by chance. Although the family type variable was retained (*p* < .05), and the model obtained was significant (omnibus test χ2 = 6.64, *p* = 0.03), with an adequate adjustment (Hosmer-Lemeshow χ2 = 0.001, *p* > 0.05), other statistics were unsatisfactory. The variance explained was minimal (Nagelkerke’s *R*^2^ = 0.02), as was the percentage of correctly classified cases (54.9%). The family type variable met the established criterion for significance (Table 4).

**Table 4:** Predictors of the profile of family caregivers of children with chronic diseases

	OR	CI 95%	*p*
Family type			
Single parent	1.69	0.91–3.15	0.09
Nuclear	0.79	0.51–1.22	0.29
Other (R)			

*R* reference category, *OR* odds ratio, *CI* confidence interval

## Discussion

The objectives of this study were to identify the psychosocial profiles of caregivers of pediatric patients with chronic disease and to establish the relationship between these profiles and sociodemographic variables. The results identified two profiles among the analyzed caregivers. Profile 1, *Vulnerability of family caregivers*, is characterized by caregivers with low levels of family support, resilience and well-being and high levels of anxiety, overload, depression and parental stress. This profile identifies caregivers at risk of presenting psychopathologies that affect the quality of the care they provide to the pediatric patient. Profile 2, *Adversity of family caregivers*, includes caregivers who show high levels of family support, resilience and well-being, which may be the reason for their low levels of anxiety, overload, depression and parental stress. Profile 2 showed that the characteristics of family caregivers can improve the quality of the care offered to the pediatric patient and contribute to the clinical improvement of the patient’s condition or at least avoid adverse changes due to factors other than the patient’s medical condition.

The sociodemographic variables were similar between both profiles. Most caregivers were mothers with basic schooling who were homemakers, professed some religious belief and had a low income. These results are consistent with the profile identified in Latin America and the Caribbean, where long-term care is generally performed by women, as required by the multiplicity of personal and family demands [1, 2, 8, 46]. Similarities between the profiles were also observed for the pediatric patient’s variables. The pediatric patient for whom the caregivers are responsible is usually a minor aged 10 years whose condition was diagnosed in the last year and whose duration of hospitalization does not exceed 1 week. The diagnosis of the pediatric patients is mostly some type of cancer, which has a profound impact on the patient’s caregiver due to the emotional consequences and the long duration of treatment [2, 47].

An important difference between profiles is that Profile 1 includes a greater number of caregivers from a single-parent family. However, the strength of the association between the family type and the caregiver’s membership cluster was low, which implies that this result has little practical relevance. Consistent with this interpretation, the family type variable did not reach statistical significance in the elaborated regression model, although it clarified the greater difficulties and adverse consequences to which single-parent caregivers are exposed [47, 48]. Therefore, future studies should delve deeper into the role that the type of family (single parent vs. nuclear) plays in the profile of caregivers of pediatric patients.

The identified profiles show similarities in the sociodemographic variables of the caregiver and the pediatric patient. This similarity extends to the clinical variables of the pediatric patient. In addition, the only variable associated with a specific profile (i.e., family type) lacks practical relevance and predictive utility. This set of results does not allow us to clearly identify what defines one or another cluster, although the two clusters are clearly opposed in terms of their psychological vulnerability. One factor that may explain this situation is the level of family support reported through the FSQ. Cluster 2 obtained higher scores on this instrument; the effect size was large, which implies significant practical relevance, and is categorized as adequate family support. Thus, the perception of adequate family support can be a factor that decreases the levels of anxiety, depression, burden and parental stress in caregivers while promoting resilience and well-being. Although this interpretation should undergo empirical verification, it is consistent with previous studies that have emphasized the central role of the family in Mexican culture [1, 2, 8], the influence of the dynamics of intrafamily relationships during care [20] and the importance of family support on the well-being of the caregiver [2].

It is important to mention that a high percentage of caregivers in each cluster lacked paid work. It is possible that the family support provided to the caregivers in cluster 2 included financial support. If so, this could also explain the differences in psychological vulnerability between the two clusters. Future studies should delve into the type of family support received by Mexican caregivers of pediatric patients.

Although there are more women than men in the caregiving role, the results of this study indicate that there are no differences in the profiles of mothers and fathers who provide care for the pediatric patient. One possible interpretation of this fact is that it is more common for women to look after a child. By assuming the caregiver role, both parents are presented with similar experiences and challenges, and the different adaptive outcomes are related to psychosocial aspects more than to demographic features per se [49].

Another striking feature of the sample studied is that the patients’ mothers and fathers were the caregivers in all cases, so the type and quality of the parental link could exert a moderating effect on the caregivers’ adaptive results. Future studies comparing different family members (e.g., grandmothers, aunts, uncles) and levels of attachment could help to confirm this hypothesis [50]. Similarly, longitudinal research designs could identify the effects of time on the care experience [51].

Among the limitations of this study is the lack of a representative sample, which limits the generalization of the data. Future studies should verify the persistence of these profiles to establish their consistency among the Mexican population of caregivers for pediatric patients.

The practical implications of this study suggest that the theoretical, practical, social and methodological importance of determining the caregiver’s profile to account for both psychosocial and sociodemographic factors substantially contributes to research on families of children with chronic diseases by helping to generate measurement, assessment and intervention programs to reduce the impact of the disease, its psychosocial effects, the consequences of care and caregiver burnout [52, 53].

## Conclusions

Previous studies have emphasized the importance of studying the impact of sociodemographic and psychosocial variables on the role of family caregivers and their adjustment to their children’s disease and treatment. However, most studies address the two perspectives separately, thus offering intervention alternatives that are not comprehensive. This research offers an interesting perspective by presenting a comprehensive approach to the sociodemographic and psychosocial factors that constitute the caregiver profile in contexts of adversity resulting from pediatric disease. In this sense, the characterization of the family caregiver results from the continual interaction of personal, sociocultural and family factors and the strength required to confront and overcome the disease. One of the strengths with the greatest positive impact on the caregiver is family support, which contributes to positive adaptation during the diagnosis and long-term treatment of the child.

## Data Availability

The set of data supporting the conclusions of this publication is included within the article.

## Abbreviations

M: Mean
SD: Standard deviation
USD: United States Dollars
